# Changes in donor lymphocyte infusion for relapsed patients post-hematopoietic stem cell transplantation: a 30-year single-center experience

**DOI:** 10.3389/fimmu.2025.1521895

**Published:** 2025-01-29

**Authors:** Yusuke Uchibori, Shuhei Kurosawa, Yuho Najima, Kyoko Haraguchi, Daichi Sadato, Chizuko Hirama, Yasutaka Sadaga, Kaori Kondo, Chika Kato, Satoshi Sakai, Yasuhiro Kambara, Fumihiko Ouchi, Masashi Shimabukuro, Atsushi Jinguji, Naoki Shingai, Takashi Toya, Hiroaki Shimizu, Takeshi Kobayashi, Hironori Harada, Yuka Harada, Yoshiki Okuyama, Noriko Doki

**Affiliations:** ^1^ Hematology Division, Tokyo Metropolitan Cancer and Infectious Diseases Center, Komagome Hospital, Tokyo, Japan; ^2^ Department of Transfusion and Cell Therapy, Tokyo Metropolitan Cancer and Infectious Diseases Center, Komagome Hospital, Tokyo, Japan; ^3^ Clinical Research and Trials Center, Tokyo Metropolitan Cancer and Infectious Diseases Center, Komagome Hospital, Tokyo, Japan; ^4^ Laboratory of Oncology, School of Life Sciences, Tokyo University of Pharmacy and Life Sciences, Tokyo, Japan

**Keywords:** donor lymphocyte infusion, hematopoietic stem cell transplantation, refined disease risk index, acute graft-versus-host disease, acute myeloid leukemia, myelodysplastic syndromes, azacitidine, venetoclax

## Abstract

**Introduction:**

Donor lymphocyte infusion (DLI) is a therapeutic approach for relapse after hematopoietic stem cell transplantation (HSCT). Despite their reported efficacy, the evolution of DLI practices over time remains underexplored.

**Methods:**

This study provided a comprehensive analysis of DLI strategies and outcomes over 30 years at a single institution. A retrospective analysis was conducted on 75 patients who underwent DLI for disease relapse between April 1994 and March 2024. The primary endpoint was the 3-year overall survival (OS) rate after DLI. Secondary endpoints included the 100-day complete remission (CR) rate and incidence of acute graft-versus-host disease (GVHD).

**Results:**

The median age at the first DLI was 49 years (range, 20–69 years). The most common underlying diseases in all 75 cases were acute myeloid leukemia (AML, n = 46) and myelodysplastic syndromes (MDS, n = 12). Until 2014, DLI was only performed in patients with AML (n = 14), MDS (n = 2), or chronic myeloid leukemia (n = 5). However, since 2015, patients with various diseases, including lymphoid malignancies, have also undergone DLI. Azacitidine was the most frequently used combination therapy with DLI (n = 34). Regimens including venetoclax and FLT3 inhibitors have been commonly used since 2019 (n = 18). The 3-year OS rate was 29.1% (95% CI, 18.8–40.2%). Factors negatively influencing OS included age ≥50 years and a high or very high refined disease risk index. The 100-day CR rate was 52.1%, and acute GVHD occurred in 25.3% of the patients, with no strong correlation between GVHD incidence and CR achievement. Among 18 patients who underwent three or more DLIs since 2018, 88.9% achieved remission following DLI or second HSCT, with a median follow-up of 949.5 days for survivors.

**Conclusion:**

This study highlighted the evolving trends in DLI practices and the diversification of combination therapies. Future research should focus on further validating these findings and optimizing DLI protocols to improve patient outcomes.

## 1 Introduction

Hematopoietic stem cell transplantation (HSCT) remains the cornerstone of treatment for high-risk hematological disorders and offers a potential cure. Despite improvements in non-relapse mortality rates, relapse remains a significant challenge to be addressed (1, 2). Donor lymphocyte infusion (DLI) is a promising therapeutic approach for post-transplant relapse (3–8). Historically, DLI has shown efficacy in treating chronic myeloid leukemia (CML) (9–11). However, the advent of tyrosine kinase inhibitors (TKIs) has led to a decline in the use of HSCT for CML (12, 13). In cases of acute myeloid leukemia (AML) and myelodysplastic syndromes (MDS), achieving a graft-versus-leukemia (GVL) effect is more challenging because of the lower expression of costimulatory and adhesion molecules than that in CML (14, 15). The rapid progression of these diseases often necessitates therapies beyond DLI (16). In addition, the use of DLI in lymphoid malignancies is less frequently reported than in myeloid malignancies (17, 18).

Over the past decade, the diversity of donor sources has expanded, with an increasing number of transplants performed using haploidentical donors in addition to human leukocyte antigen (HLA)-matched donors (19, 20). Furthermore, the advent of targeted molecular therapies, such as BCL2 and FLT3 inhibitors, has broadened the treatment options for refractory AML cases (21–23). Similarly, the emergence of bispecific antibodies and chimeric antigen receptor T-cell therapy has significantly transformed the therapeutic approach to lymphoid malignancies (24–26). In this era of complex therapeutic regimens, the effect of DLI on contemporary HSCT practices and outcomes remains unclear. Thus, this study aimed to provide a comprehensive analysis of post-transplant DLI cases over the past 30 years at a single institution, evaluate the evolution of DLI strategies, and identify the prognostic factors influencing outcomes in the current therapeutic context.

## 2 Patients and methods

### 2.1 Ethical approval and study population

We retrospectively analyzed patients with hematological malignancies who underwent DLI for disease relapse at our center between April 1994 and March 2024. The final day of observation was July 21, 2024. This study was approved by the Institutional Research Ethics Board of Tokyo Metropolitan Komagome Hospital (approval number: 2741) and was performed according to the tenets of the Declaration of Helsinki. Informed consent was obtained from the website in the form of opt-out.

### 2.2 Transplantation procedures

The classification of myeloablative and reduced-intensity conditioning regimens was predicated on a prior publication (27). For HLA-matched or single-locus mismatched HSCT, myeloablative conditioning predominantly encompassed a total body irradiation (TBI) protocol (12 Gy), incorporating cyclophosphamide (CY; 60 mg/kg for 2 days) or a non-TBI regimen comprising intravenous busulfan (ivBU; 3.2 mg/kg for 4 days), and either CY (60 mg/kg for 2 days) or fludarabine (FLU; 180 mg/m^2^). Reduced-intensity conditioning primarily consisted of FLU (30 mg/m^2^ for 6 days), either ivBU (3.2 mg/kg for 2 days) or melphalan (40 or 70 mg/m^2^ for 2 days), and TBI (4 Gy). We implemented a calcineurin inhibitor (cyclosporine or tacrolimus) augmented with short-term methotrexate for graft-versus-host disease (GVHD) prophylaxis. Rabbit anti-thymocyte globulin (rATG) was added for GVHD prophylaxis at the discretion of the attending physician (28).

Haploidentical donors were defined as related donors exhibiting a 4/8 to 6/8 match at the allele level for HLA-A, HLA-B, HLA-C, and HLA-DRB1. The conditioning regimens and GVHD prophylaxis protocols for haploidentical HSCT encompassed a regimen incorporating low-dose rATG and an alternative protocol utilizing post-transplant CY, with the selection guided by the attending physician’s discretion. These methodologies have been elucidated in previous studies (29, 30).

### 2.3 Study endpoints and definitions

The primary endpoint was the 3-year overall survival (OS) rate after DLI. The secondary outcomes were the 100-day complete remission (CR) rate and incidence of acute GVHD after the first DLI. We defined CR as the complete disappearance of all clinical, radiological, and histological/immunophenotypic evidence as described in a previous study (6). Disease risk classification was divided into “low,” “intermediate,” “high,” and “very high” using the refined disease risk index (R-DRI) at the time of HSCT (31). Previously established criteria were used to diagnose and grade acute and chronic GVHD (32, 33).

### 2.4 Statistical analyses

OS was estimated using the Kaplan–Meier method, and the stratified comparisons between groups were conducted using the log-rank test. The incidence of acute GVHD after DLI was evaluated using Gray’s method, with death and receiving subsequent HSCT considered as competing risk factors. To elucidate prognostic factors influencing OS, both univariate and multivariate analyses were conducted using the Cox proportional hazards regression model. We introduced factors with a P-value < 0.20 in the univariate analysis into the multivariate analysis. Hazard ratios (HRs) and 95% confidence intervals (CI) were estimated using the Cox regression model. All statistical tests were 2-sided, with P values less than.05 considered statistically significant. All statistical analyses were performed with EZR (Saitama Medical Center, Jichi Medical University, Saitama, Japan), which is a graphical user interface for R (The R Foundation for Statistical Computing, Vienna, Austria, version 4.4.1). More precisely, it is a modified version of R commander (version 1.68) designed to add statistical functions frequently used in biostatistics (34).

## 3 Results

### 3.1 Baseline characteristics of the study population

**Figure 1:**
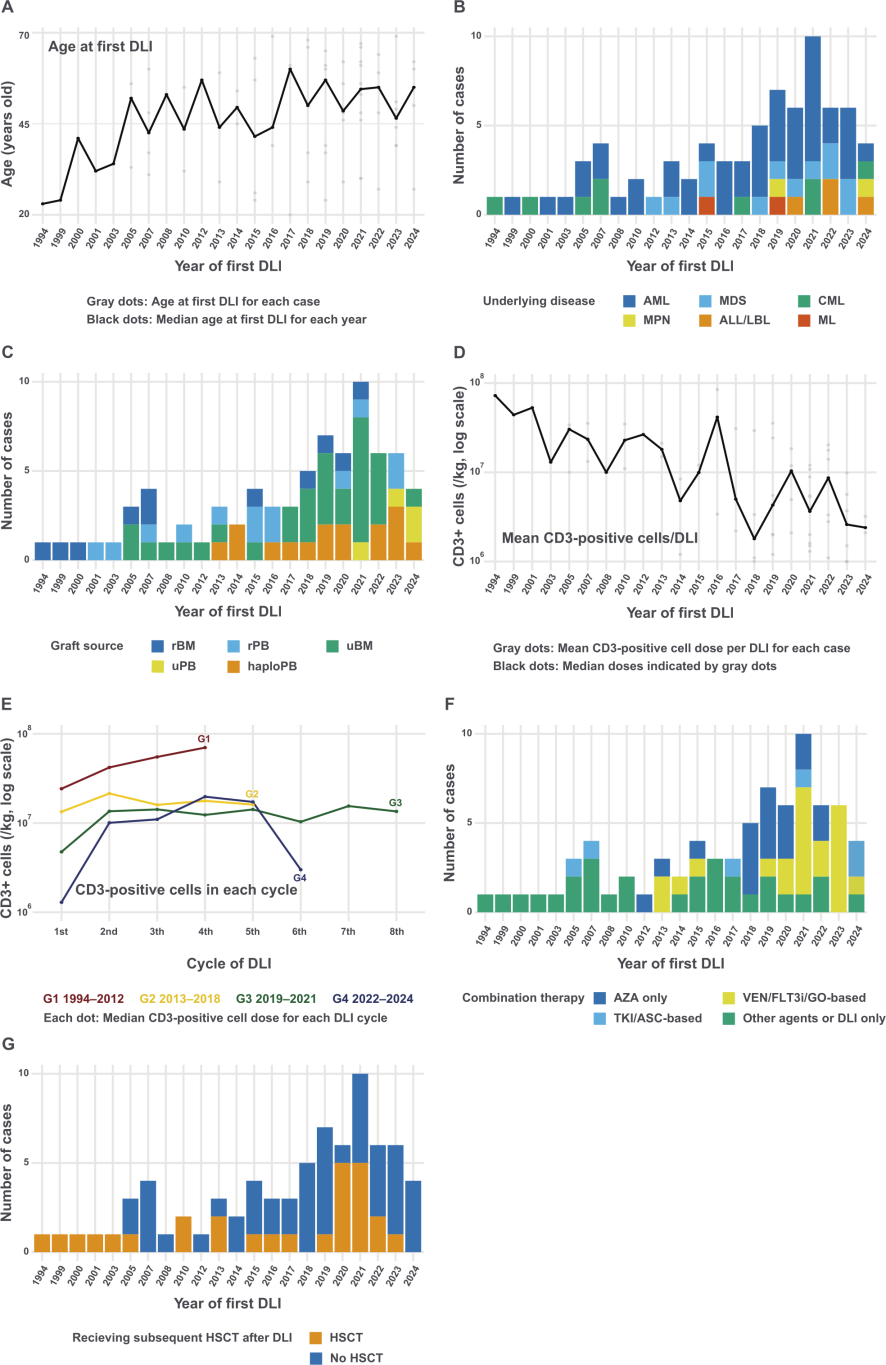
Changes in donor lymphocyte infusion at our institution **(A)** Median age at the first DLI by year. The plot shows the median age of patients receiving DLI each year, with individual ages represented by gray dots and the median age connected by a black line. **(B)** Number of DLI cases by year and underlying disease. **(C)** Number of DLI cases by year and graft source. **(D)** Temporal trend of mean CD3-positive cell doses (log scale). Gray dots indicate the mean CD3-positive cell dose per DLI for individual cases, while black dots represent the median of these means for each year. **(E)** Pattern of CD3-positive cell doses across DLI cycles by year of first DLI (log scale). Lines represent different time periods (Group [G] 1: 1994–2012, G2: 2013–2018, G3: 2019–2021, G4: 2022–2024), showing the median CD3-positive cell dose for each DLI cycle within each period. **(F)** Number of DLI cases by year and combination treatment. Regimens including venetoclax, FLT3 inhibitors, and gemtuzumab ozogamicin were classified into VEN/FLT3i/GO-based. Regimens including tyrosine kinase inhibitors and asciminib were classified into TKI/ASC-based. **(G)** Number of DLI cases by year and subsequent HSCT. ALL, acute lymphoblastic leukemia; AML, acute myeloid leukemia; ASC, asciminib; AZA, azacitidine; CML, chronic myeloid leukemia; DLI, donor lymphocyte infusion; FLT3i, FLT3 inhibitor; GO, gemtuzumab ozogamicin; haploPB, haploidentical peripheral blood; HSCT, hematopoietic stem cell transplantation; LBL, lymphoblastic lymphoma; MDS, myelodysplastic syndromes; ML, malignant lymphoma; rBM, related bone marrow; rPB, related peripheral blood; TKI, tyrosine kinase inhibitor; uBM, unrelated bone marrow; uPB, unrelated peripheral blood.

The patient characteristics are summarized in **Table 1** and **Figure 1**. At our institution, a total of 2,018 allogeneic or syngeneic HSCTs were performed between 1992 and 2023, with 510 patients experiencing relapse. Among these, 75 patients (14.7%) underwent DLI. The median age of the patients at the first DLI was 49 years (range: 20–69 years). The median age gradually increased over the study period, with older patients receiving DLI in recent years (**Figure 1A**). Until 2014, Among the underlying diseases, AML (n = 14) and CML (n = 5) were predominant until 2014. However, after 2015, patients with various diseases, including lymphoid malignancies, began to undergo DLI (**Figure 1B**). Thirty patients had low or intermediate R-DRI values. The numbers of HSCT before DLI were 1 (n = 59, 78.7%), 2 (n = 15, 20%), and 3 (n = 1, 1.3%). Among the graft sources, 24 patients (32.0%) received HLA-matched related donors (MRD), 35 (46.7%) received unrelated donors (UD), and 16 (21.3%) were administered HLA-haploidentical donors (Haplo). Since 2013, DLI has been performed on haploidentical donors and, since 2021, on unrelated peripheral blood stem cell donors (**Figure 1C**).

**Table 1:** Baseline characteristics of the study population.

Patient characteristics		N (%)
Age at first DLI	Years, median [range]	49 [20–69]
Sex	Female	29 (38.7)
	Male	46 (61.3)
Underlying disease	AML	46 (61.3)
	MDS	12 (16.0)
	CML	9 (12.0)
	MPN	2 (2.7)
	ALL/LBL	4 (5.3)
	ML	2 (2.7)
R-DRI at HSCT	Low	7 (9.3)
	Intermediate	23 (30.7)
	High	37 (49.3)
	Very high	8 (10.7)
Year of HSCT	Years, median [range]	2018 [1992–2023]
Numbers of HSCT before DLI	1	59 (78.7)
	2	15 (20.0)
	3	1 (1.3)
Graft source	HLA-matched related BM	11 (14.7)
	HLA-matched related PBSC	13 (17.3)
	Unrelated BM	31 (41.3)
	Unrelated PBSC	4 (5.3)
	HLA-haploidentical related PBSC	16 (21.3)
Conditioning intensity	Myeloablative	42 (56.0)
	Reduced intensity	33 (44.0)
Interval from HSCT to relapse	Days, median [range]	178 [21–2,688]
Interval from relapse to first DLI	Days, median [range]	49 [7–443]
Relapse type	Hematological relapse	32 (42.7)
	Extramedullary relapse	8 (10.7)
	Molecular or cytogenetic relapse	35 (46.7)
Numbers of DLI	1	30 (40.0)
	2	21 (28.0)
	≥3	24 (32.0)
Infused CD3-positive cells	Initial dose, ×10^7^ cells/kg, median [range]	0.43 [0.06–8.45]
	Mean dose per DLI, ×10^7^ cells/kg, median [range]	0.99 [0.06–8.45]
	Total dose, ×10^7^ cells/kg, median [range]	1.06 [0.06–29.0]
Combination therapy with DLI	Azacitidine only	18 (24.0)
	Azacitidine and venetoclax	10 (13.3)
	Azacitidine and FLT3 inhibitor	1 (1.3)
	Azacitidine and gemtuzumab ozogamicin	4 (5.3)
	Azacitidine, venetoclax, and FLT3 inhibitor	1 (1.3)
	FLT3 inhibitor only	5 (6.7)
	Venetoclax only	1 (1.3)
	TKI only	3 (4.0)
	TKI and other chemotherapy	2 (2.7)
	Asciminib	1 (1.3)
	Other cytotoxic chemotherapies only	16 (21.3)
	Steroid, interferon, radiotherapy, or tretinoin	5 (6.7)
	DLI only	8 (10.7)
Receiving subsequent HSCT after DLI	Yes	27 (36.0)
	No	48 (64.0)
Year of first DLI	Years, median [range]	2018 [1994–2024]

ALL, acute lymphoblastic leukemia; AML, acute myeloid leukemia; BM, bone marrow; CML, chronic myeloid leukemia; DLI, donor lymphocyte infusion; HLA, human leukocyte antigen; HSCT, hematopoietic stem cell transplantation; LBL, lymphoblastic lymphoma; MDS, myelodysplastic syndromes; ML, malignant lymphoma; MPN, myeloproliferative neoplasms; N, number; PBSC, peripheral blood stem cells; R-DRI, refined disease risk index; TKI, tyrosine kinase inhibitor.

The median interval from HSCT to relapse was 178 days (range, 21–2,688 days). The relapse types were hematological (n = 32, 42.7%), extramedullary (n = 8, 10.7%), or molecular/cytogenetic (n = 35, 46.7%). **Figure 1D** displays the mean CD3-positive cell dose infused per DLI for each case, with the doses showing a decreasing trend over the years. **Figure 1E** demonstrates the CD3-positive cell doses across each DLI cycle, stratified by the year of the first DLI. The initial doses show a decreasing trend in more recent periods, particularly in patients receiving from Haplo (**Supplementary Figure 1**). Azacitidine was the most frequently used combination therapy with DLI (n = 34, 45.3%). Regimens including venetoclax and FLT3 inhibitors have been commonly used since 2019 (n = 18, 24.0%; **Figure 1F**). Twenty-seven (36.0%) patients underwent HSCT after DLI (**Figure 1G**).

**Supplementary Table 1** shows baseline characteristics stratified by year of DLI. The first group consisted of 36 cases from 1994 to 2018, and the second group included 39 cases from 2019 to 2024. Patients in the 2019–2024 cohort tended to be older than those in the 1994–2018 cohort (P = 0.12), had a greater proportion of HSCT from unrelated or Haplo donors (P = 0.042), received a lower CD3-positive cell dose per DLI (P < 0.001), and had a higher frequency of concurrent chemotherapy with DLI (P = 0.006).

### 3.2 DLI outcomes and prognostic factors

**Figure 2:**
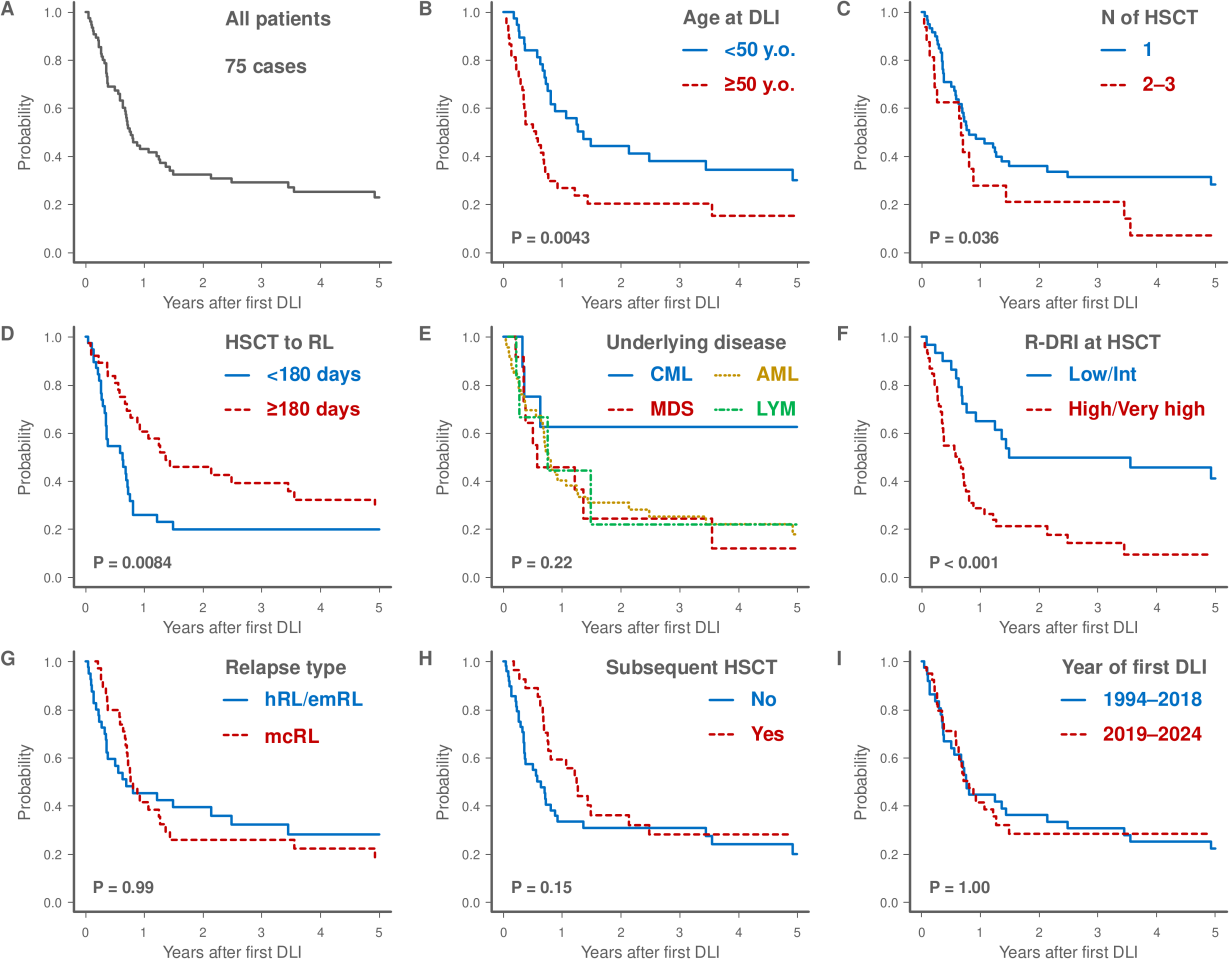
Kaplan-Meier survival curves of the 75 patients who underwent DLI **(A)**. The plots show overall survival stratified by various factors and the log-rank test was used to assess any significant differences: age at first DLI (<50 versus ≥50 years), **(B)**, number of HSCT before DLI (1 versus 2–3), **(C)**, interval from HSCT to relapse (<180 days versus ≥180 days), **(D)**, underlying disease (CML versus MDS versus AML versus LYM), **(E)**, R-DRI at HSCT (Low/Intermediate versus High/Very high), **(F)**, relapse type (hRL/emRL versus mcRL), **(G)**, receiving subsequent HSCT (Yes versus No, **H**), and year of first DLI (1994–2018 versus 2019–2024), **(I)**. AML, acute myeloid leukemia; CML, chronic myeloid leukemia; DLI, donor lymphocyte infusion; emRL, extramedullary relapse; hRL, hematological relapse; HSCT, hematopoietic stem cell transplantation; LYM, lymphoid malignancies; mcRL, molecular or cytogenetic relapse; MDS, myelodysplastic syndromes; N, number; R-DRI, refined disease risk index; RL, relapse.

The median follow-up period from first DLI for survivors was 1,157 days (range, 104–10,869 days). Regarding the study endpoints, the 3-year OS rate after DLI was 29.1% (95% confidence interval [CI], 18.8–40.2%; **Figure 2A**). The 3-year OS rate was significantly higher in patients aged <50 years than that in those aged ≥50 years (38.0% [95% CI, 22.2–53.6%] versus 20.3% [95% CI, 8.8–35.1%]; **Figure 2B**). Patients who had undergone a single HSCT prior to DLI had better outcomes than those who had undergone two or three HSCTs (31.3% [95% CI, 19.4–43.9%] versus 20.8% [95% CI, 5.2–43.6%]; **Figure 2C**). The relapse interval of ≥180 days post-HSCT was associated with improved OS compared to the relapse interval of <180 days (39.1% [95% CI, 22.9–55.0%] versus 19.6% [95% CI, 8.5–34.2%]; **Figure 2D**). The 3-year OS rate for patients with CML was 62.5% (95% CI, 22.9–86.1%), which was higher than that of other subtypes, including AML (25.4% [95% CI, 13.5–39.2%]), MDS (24.4% [95% CI, 4.5–52.8%]), and lymphoid malignancies (22.2% [95% CI, 1.0–61.5%]) (**Figure 2E**). Patients with low or intermediate R-DRI demonstrated better 3-year OS than those with high or very high R-DRI (49.7% [95% CI, 30.1–66.5%] versus 14.1% [95% CI, 5.1–27.5%]; **Figure 2F**). Relapse type, subsequent HSCT, and year of DLI did not significantly affect the OS (**Figures 2G–I**).

The results of univariate analysis evaluating the pre-DLI prognostic factors for OS are presented in **Supplementary Table 2**. In the multivariate analysis, patients aged ≥50 years (HR 3.10; 95% CI, 1.74–5.53; P < 0.001) and those with high or very high R-DRI (HR 3.45; 95% CI, 1.72–6.92; P < 0.001) were identified as adverse prognostic factors for OS (**Table 2**).

**Table 2:** Multivariate analysis for overall survival in the entire cohort.

Factor	Group	N	HR (95% CI)	*P*
Age at first DLI	<50 years	38	1	
	≥50 years	37	3.10 (1.74–5.53)	<0.001
R-DRI	Low or Intermediate	30	1	
	High or Very high	45	3.45 (1.72–6.92)	<0.001
Numbers of HSCT before DLI	1	59	1	
	2 or 3	16	1.52 (0.82–2.80)	0.18
Interval from HSCT to relapse	<180 days	38	1	
	≥180 days	37	0.70 (0.39–1.27)	0.24

DLI, donor lymphocyte infusion; HR, hazard ratio; HSCT, hematopoietic stem cell transplantation; N, number; R-DRI, refined disease risk index.

### 3.3 Subgroup analyses in patients with acute myeloid leukemia and myelodysplastic syndromes

A subgroup analysis focusing on AML (n = 46) and MDS (n = 12) was performed, and the patient characteristics are summarized in **Supplementary Table 3**. In the AML subgroup, the median age at the first DLI was 47 years (range: 20–69 years). Seventeen patients (37.0%) received subsequent HSCT after DLI. The median OS was 273 days (range: 18–9281 days). Stratified analysis using the log-rank test revealed that 3-year OS was significantly inferior in patients aged ≥50 years compared to those aged <50 years (not calculable vs. 40.9% [95% CI, 21.8–59.1%]) and in those with high or very high R-DRI compared to those with low or intermediate R-DRI (18.2% [95% CI, 6.6–34.5%] vs. 41.7% [95% CI, 15.2–66.5%]; **Supplementary Figure 2**). Univariate Cox proportional hazards analysis identified age ≥50 years (HR 4.37; 95% CI, 2.09–9.12; P < 0.001) and high or very high R-DRI (HR 2.26; 95% CI, 1.01–5.07; P = 0.049) as adverse prognostic factors for OS (**Supplementary Table 4**).

In the MDS subgroup, the median age at the first DLI was 58 years (range: 26–69 years), and all but one patient received azacitidine in combination with DLI (**Supplementary Table 2**). Only two patients (16.7%) received subsequent HSCT after DLI. The median OS was 198 days (range: 78–2,736 days). Stratified analysis using the log-rank test demonstrated significantly worse 3-year OS in patients with relapse intervals <180 days compared to those with relapse intervals ≥180 days (not calculable vs. 44.4% [95% CI, 6.6–78.5%]) and in those with high or very high R-DRI compared to those with low or intermediate R-DRI (not calculable vs. 53.3% [95% CI, 6.8–86.3%]; **Supplementary Figure 3**). Univariate Cox analysis identified a relapse interval ≥180 days (HR 0.14; 95% CI, 0.03–0.77; P = 0.024) as a favorable prognostic factor and high or very high R-DRI (HR 10.54; 95% CI, 1.22–91.24; P = 0.033) as an adverse prognostic factor for OS (**Supplementary Table 5**).

### 3.4 Impact of acute GVHD on outcomes after DLI

**Figure 3:**
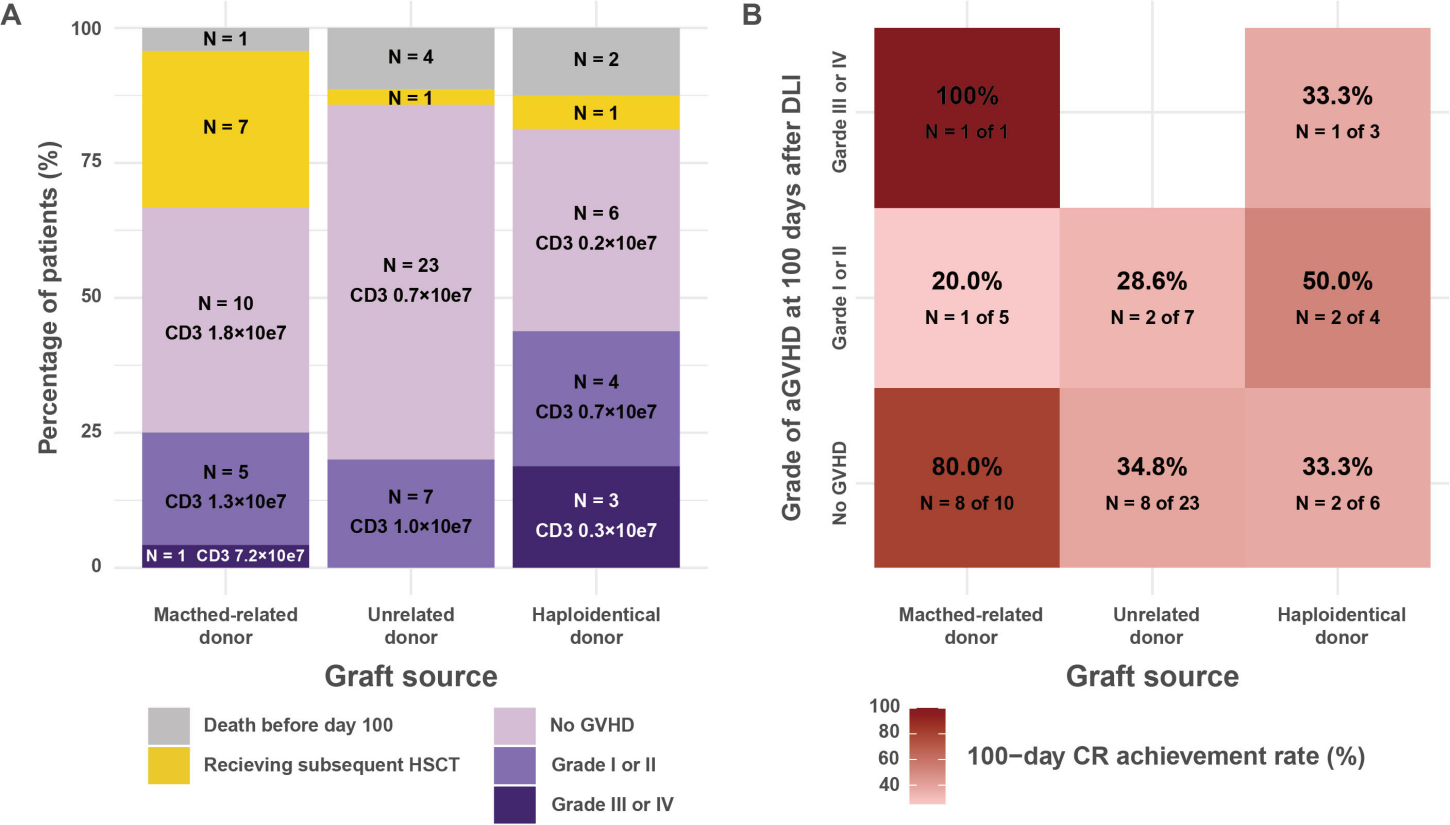
Acute GVHD incidence and CR rates following donor lymphocyte infusion stratified by graft source. **(A)** Maximum grade of acute GVHD at 100 days post-DLI, categorized by donor source. The bar graph displays the percentage of patients with varying severities of acute GVHD (death before day 100, subsequent HSCT, no GVHD, grades I or II, and grades III or IV) after DLI. Death before day 100 and subsequent HSCT were treated as competing events for the development of acute GVHD. The data are stratified by donor source into matched-related, unrelated, and haploidentical donors. Each bar indicates the number and percentage of patients, along with the median CD3-positive cell dose (average dose per DLI). **(B)** Heatmap depicting the 100-day CR rates, stratified by donor source and acute GVHD severity. The color intensity corresponds to the CR rate, with darker shades indicating higher rates. Each cell contains the CR rate as a percentage, along with the number of patients achieving CR out of the total in that category (N). The analysis excludes patients who died before day 100. CR rates are shown for patients without GVHD, with grade I or II GVHD, and with grade III or IV GVHD, across the three donor sources. CR, complete remission; DLI, donor lymphocyte infusion; GVHD, graft-versus-host disease; HSCT, hematopoietic stem cell transplantation.

The 100-day CR rate and incidence of acute GVHD were 52.1% and 25.3%, respectively. **Figure 3A** illustrates the severity of acute GVHD at 100 days post-DLI, stratified by graft source. This figure also includes patients who died within 100 days and those who underwent subsequent HSCT. Acute GVHD was observed in 6 patients with MRD, 7 with UD, and 7 with Haplo. No GVHD-related deaths occurred following DLI.

**Figure 3B** presents a heatmap depicting the 100-day CR achievement rates, categorized by graft source and acute GVHD severity. Among patients without acute GVHD, the 100-day CR rates were 80.0% (8 of 10 patients) for MRD, 34.8% (8 of 23) for UD, and 33.3% (2 of 6) for Haplo. In cases of grade I to II acute GVHD, the CR rates were 20.0% (1 of 5) for MRD, 28.6% (2 of 7) for UD, and 50.0% (2 of 4) for Haplo. In patients with grade III to IV acute GVHD, the CR rates were 100.0% (1 of 1) for MRD and 33.3% (1 of 3) for Haplo.

### 3.5 Detailed characteristics of recent DLI cases

**Figure 4:**
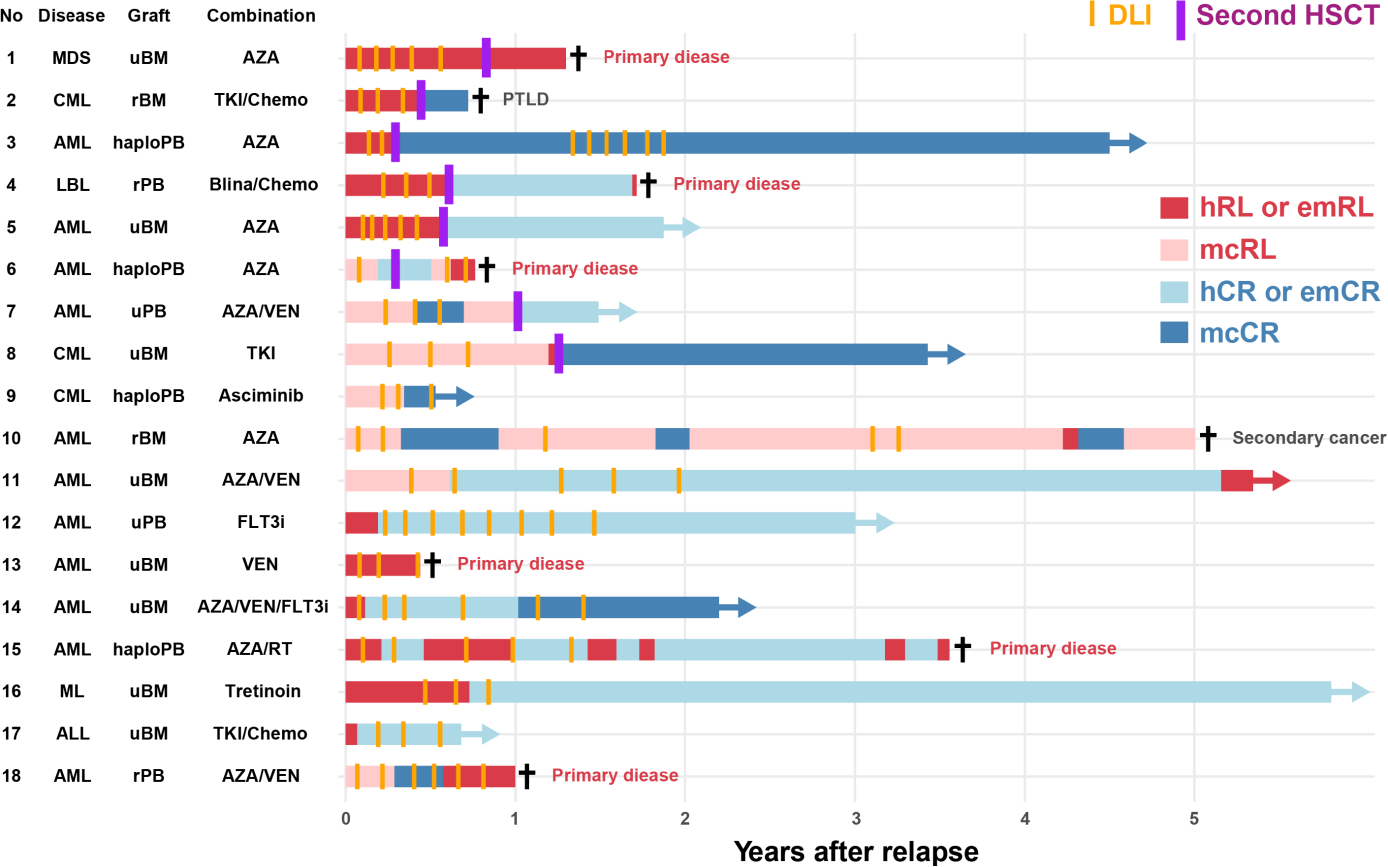
Swimmer plot of recent cases receiving donor lymphocyte infusion This swimmer plot illustrates the clinical course of 18 patients who received donor lymphocyte infusions three or more times since 2018. Horizontal bars represent the duration from relapse to subsequent clinical events, such as DLI and second HSCT. Colors indicate different clinical states and therapies, with markers denoting significant events, such as survival and death. ALL, acute lymphoblastic leukemia; AML, acute myeloid leukemia; ASC, asciminib; AZA, azacitidine; Blina, blinatumomab; Chemo, chemotherapy; CML, chronic myeloid leukemia; DLI, donor lymphocyte infusion; emCR, complete remission; emRL, extramedullary relapse; FLT3i, FLT3 inhibitor; haploPB, haploidentical peripheral blood; hCR, hematological complete remission; hRL, hematological relapse; HSCT, hematopoietic stem cell transplantation; LBL, lymphoblastic lymphoma; mcCR, molecular or cytogenetic complete remission; mcRL, molecular or cytogenetic relapse; MDS, myelodysplastic syndromes; ML, malignant lymphoma; PB, peripheral blood; PTLD, post-transplant lymphoproliferative disorder; rBM, related bone marrow; rPB, related peripheral blood; RT, radiotherapy; TKI, tyrosine kinase inhibitor; uBM, unrelated bone marrow; uPB, unrelated peripheral blood; VEN, venetoclax.

The 3-year OS for patients who received three or more DLIs was 68.0% (95% CI, 44.3–83.3%). Their median survival duration was 893 days (range, 114–10,869 days) (**Supplementary Figure 1**). **Figure 4** elucidates recent successful strategies for DLI use through a swimmer plot of 18 patients who underwent three or more DLIs since 2018. The median age at the time of the first DLI was 48 years (range, 27–66 years). The underlying diseases included AML in 11 patients (61.1%), CML in 3 patients (16.7%), lymphoid malignancies in 3 patients (16.7%), and MDS in 1 patient (5.6%). Eight patients (44.4%) underwent a second HSCT following DLI. One patient (5.6%) received prophylactic DLI after the second HSCT and maintained cytogenetic CR (case 3). Regarding combination therapies, 10 patients (55.6%) were treated with azacitidine, 5 (27.8%) with venetoclax, 3 (16.7%) with TKI, and 2 (11.1%) with FLT3 inhibitors. Case 4 treated with blinatumomab and case 12 treated with quizartinib were previously documented (35, 36). Sixteen patients (88.9%) achieved remission after either DLI or a second HSCT. Of the 10 patients who did not undergo a second HSCT, six (60.0%) were alive with a median follow-up of 949.5 days (range, 193–2,219 days) from relapse and five (50.0%) maintained remission.

## 4 Discussion

This study provides a detailed account of the 30-year evolution of DLI practices at a single institution. Recently, the patient population has aged, and the underlying diseases and combination therapies have diversified. Although limited in number, some patients achieved long-term survival without undergoing subsequent HSCT. **Table 3** presents a comparison between the results of this study and previous large-scale retrospective studies conducted in Japan (5–8). The previous studies were multi-center retrospective analyses based on registry data. Unlike HSCT, DLI lacks standardized protocols, leading to potential inter-center procedural variability. Our single-center study leveraged its strengths to provide detailed insights into DLI strategies, including CD3-positive cell dose, infusion cycles, and combination therapies, all aspects that are difficult to elucidate from registry data.

**Table 3:** Summary of large-scale retrospective studies on donor lymphocyte infusion in Japan.

Reference		Takami, et al. [5]	Miyamoto, et al. [6]	Harada, et al. [7]	Marumo et al. [8]	The present study
Study type		Registry data	Registry data	Registry data	Registry data	Single center
Year of HSCT		1991–2011	1999–2013	2006–2017	2002–2022	1992–2023
Number of cases		143	414	84	107	75
Age	Years old (range)	49 (16–67)	43 (1–78)	Therapeutic: 39 (16–64) Preemptive: 42 (16–68) Prophylactic: 32.5 (18–64)	58.5 (18–71)	49 (20–69)
Underlying disease		AML	Various diseases	AML	MDS	Various diseases
Numbers of HSCT before DLI	1 ≥2	100% 0.0%	No detailed data	36.9% 63.1%	80.4% 19.6%	78.7% 21.3%
Graft source	rBM/rPB uBM/uPB haploBM/haploPB	No detailed data	0.0% 100% 0.0%	0.0% 0.0% 100%	41.1% 38.3% 19.6%	32.0% 46.7% 21.3%
Type of relapse	hRL/emRL mcRL No RL	No detailed data	84.3% 15.7% 0.0%	42.9% 47.6% 9.5%	87.9% 12.1% 0.0%	53.3% 46.7% 0.0%
Numbers of DLI	1 2 ≥3	76.2% 15.4% 8.4%	52.9% 28.0% 19.1%	No detailed data (1–8 cycles)	No detailed data	40.0% 28.0% 32.0%
Combination therapy		No detailed data	No detailed data	GO-based: 9.5% Azacitidine: 7.1%	Azacitidine: 46.7% Venetoclax: 5.6%	See **Table 1**
Subsequent HSCT	Yes No	0% 100%	No detailed data	No detailed data	19.6% 80.4%	36.0% 64.0%
Response rate		No detailed data	CR at 100 days: 25.6%	Overall response Therapeutic: 13.9% Preemptive: 47.4%	CR at best: 30.0%	CR at 100 days: 27.1%
Grade of acute GVHD	III–IV	4.2% in total	13.3% in total	16.9% at 100 days	33.0% in total	12.5% at 100 days
Overall survival		17% at 2 years	59.7% at 100 days	13.5% at 1 year	30.0% at 1 year	29.1% at 3 years
Prognostic factors		For OS: • Days from HSCT to RL • Disease stage at DLI	For CR: • Disease status at RL • Occurrence of GVHD • CML	For OS: • Therapeutic or preemptive • Response to DLI • Days from HSCT to DLI	For OS: • Age ≥58 years • Hematologic relapse • Days from HSCT to DLI	For OS: • Age at first DLI • R–DRI
Median follow–up for survivors	Days (range)	459 (73–4,377)	No detailed data	1,122 (104–1,549)	220 (49–4,002)	1,157 (104–10,869)

AML, acute myeloid leukemia; CML, chronic myeloid leukemia; CR, complete remission; DLI, donor lymphocyte infusion; emRL, extramedullary relapse; GO, gemtuzumab ozogamicin; GVHD, graft-versus-host disease; haploBM, haploidentical bone marrow; haploPB, haploidentical peripheral blood; hRL, hematological relapse; HSCT, hematopoietic stem cell transplantation; mcRL, molecular or cytogenetic relapse; MDS, myelodysplastic syndromes; OS, overall survival; rBM, related bone marrow; R-DRI, refined disease risk index; RL, relapse; rPB, related peripheral blood; uBM, unrelated bone marrow; uPB, unrelated peripheral blood.

Studies focusing on AML have identified the interval from HSCT to relapse and disease status at the time of DLI as significant prognostic factors. In this study, subgroup analysis of AML identified age and R-DRI as significant prognostic factors. For patients with these factors, DLI may be a viable treatment option. However, these factors have also been reported as prognostic indicators in post-transplant relapse cases without DLI (2, 37, 38). Thus, further exploration is warranted to identify populations that could benefit from DLI. Additionally, with the advent of FLT3 and BCL2 inhibitors (21–23), AML treatment options have diversified, necessitating discussions on the role of DLI in the context of these emerging therapies.

In the study focusing on DLI for patients with MDS, older age and a higher prevalence of azacitidine use were frequently observed (8), trends that were also reflected in our study. For patients with MDS, post-HSCT relapse is associated with particularly poor outcomes (8, 39). Compared to those with AML, patients with MDS are often older and face greater limitations in eligibility for second or third HSCT. Moreover, treatment options beyond hypomethylating agents remain scarce. The role of DLI in patients with MDS may differ from its role in AML. To reduce the toxicity associated with both chemotherapy and cellular therapy, combining hypomethylating agents with DLI warrants further exploration as a potential treatment strategy for post-HSCT relapse in MDS (40, 41). It is crucial to build a stronger evidence base for DLI tailored specifically to patients with MDS.

In our cohort, several patients achieved long-term survival without subsequent HSCT in recent years. Whether HSCT after DLI improves patient prognosis remains inconclusive (42, 43). With the expanding array of treatment options for refractory cases, combination therapies incorporating DLI may offer a viable alternative for patients who are ineligible for a second or third HSCT. Although the efficacy and safety of combining azacitidine and/or venetoclax with DLI have been explored in several studies (40, 41, 44, 45), the present study underscores the potential for establishing the safety and efficacy of DLI in conjunction with other novel agents (35, 36, 46, 47).

This study evaluated the impact of GVHD on diverse DLI settings. Although this study included a limited number of cases, no strong correlation was observed between the incidence of GVHD and CR achievement rates. Several studies have reported that GVHD after DLI contributes to CR achievement and prolongs survival (6, 48). However, the present study included a significant percentage of patients who were administered agents other than DLI. Independent of the GVL effect, pharmacological antitumor effects may modify the incidence of GVHD and the CR achievement rates. Notably, an important finding of this study was the increasing trend in DLIs from haploidentical donors over time. In a study on DLI from haploidentical donors, the number of CD3-positive cells was associated with GVHD incidence, and severe GVHD was linked to treatment-related mortality (7). At our institution, the number of CD3-positive cells infused has shown a decreasing trend with an increase in haploidentical HSCT, and there have been no treatment-related deaths due to GVHD. With increasing treatment options for refractory cases, a safer approach may be more beneficial for DLI than the stronger GVL effect. In particular, DLI from haploidentical donors lacks sufficient evidence (7, 49, 50, 51), necessitating further investigation.

This retrospective study has several limitations. Firstly, the criteria for DLI administration, timing, CD3+ cell dose, and number of infusions were determined by each attending physician. This heterogeneity in DLI applications may have influenced our results. Second, the study only included patients who underwent DLI, necessitating cautious interpretation of the findings when generalizing to other patients. Third, the analysis combined cases with and without subsequent HSCT. The concurrent use of various medications further complicates the evaluation of the direct impact of DLI on prognosis. Fourth, this study spans a 30-year period to focus on temporal trends. This long timeframe may have introduced unaddressed factors that changed over time and influenced the outcomes. Fifth, this study comprises patients with various diseases, each receiving distinct regimens other than DLI. The heterogeneity in treatment approaches may have influenced the results. However, conducting prospective studies on post-transplant relapse remains challenging. We believe that this retrospective study offers valuable insights for both patients and healthcare providers in managing post-transplant relapse.

In conclusion, this study highlights the refined DLI strategies developed over 30 years, alongside the increasing diversity of combination therapies. Meticulous case-by-case assessments are crucial for advancing treatment, especially for patients who achieve long-term survival after DLI. Future efforts should validate these findings and optimize DLI protocols to improve outcomes.

## Data Availability

The original contributions presented in the study are included in the article/**Supplementary Material.** Further inquiries can be directed to the corresponding author.
