# Supplementary material for: Changes in donor lymphocyte infusion for relapsed patients post-hematopoietic stem cell transplantation: a 30-year single-center experience
Source: Front Immunol. 2025 Jan 29;16:1521895. doi: 10.3389/fimmu.2025.1521895 (PMC11814185; doi:10.3389/fimmu.2025.1521895)
Supplement: Supplementary file 1 [file DataSheet1.docx]

Supplementary Material

# Supplementary Figures


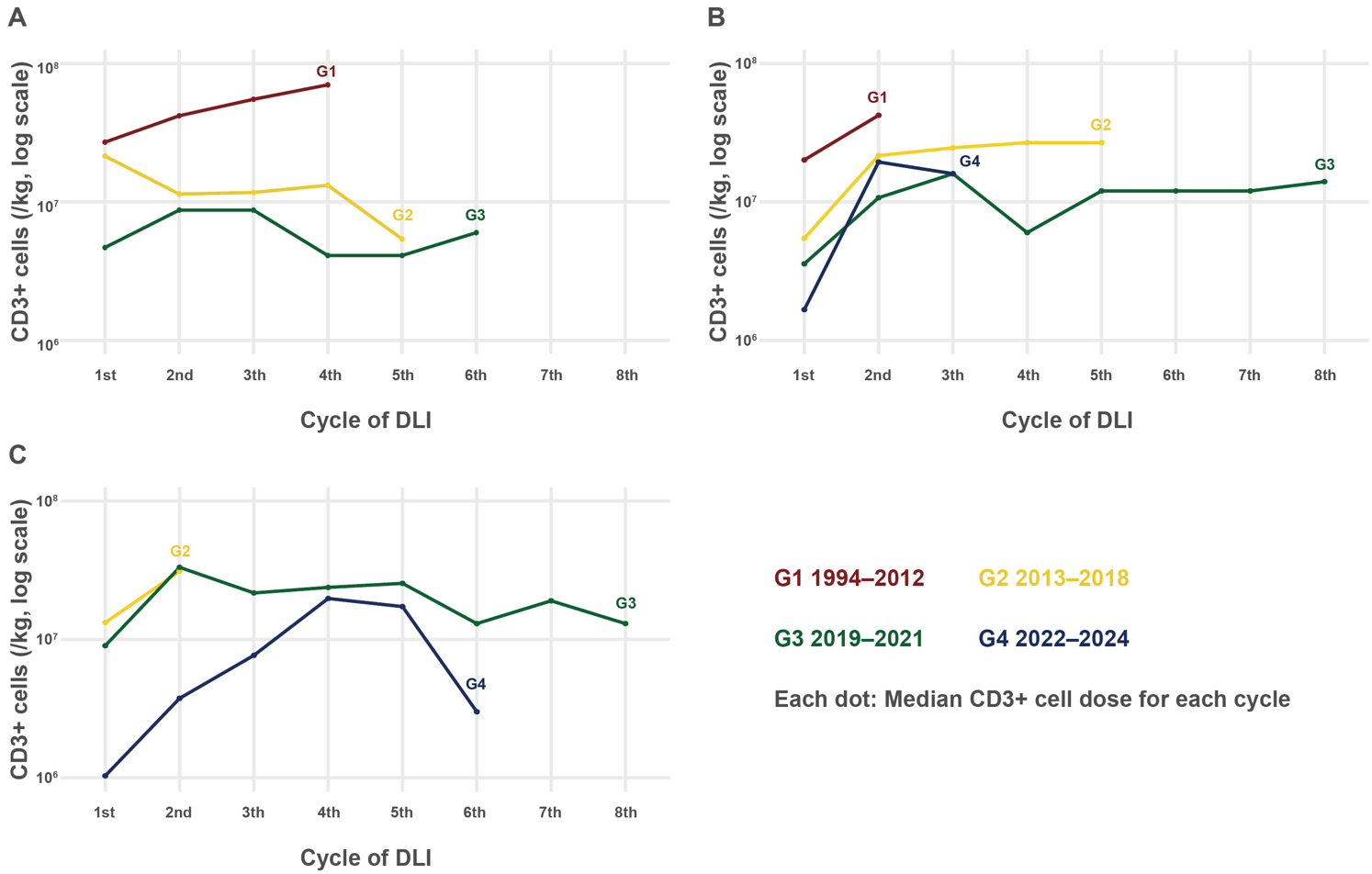


**Supplementary Figure 1. The pattern of CD3-positive cell doses across donor lymphocyte infusion cycles (log scale) stratified by graft source**

(**A**) HLA-matched related donors. (**B**) Unrelated donors. (**C**) HLA-haploidentical donors. Lines represent different time periods (Group [G] 1: 1994–2012, G2: 2013–2018, G3: 2019–2021, G4: 2022–2024), showing the median CD3-positive cell dose for each DLI cycle within each period.

DLI, donor lymphocyte infusion; HLA, human leukocyte antigen.


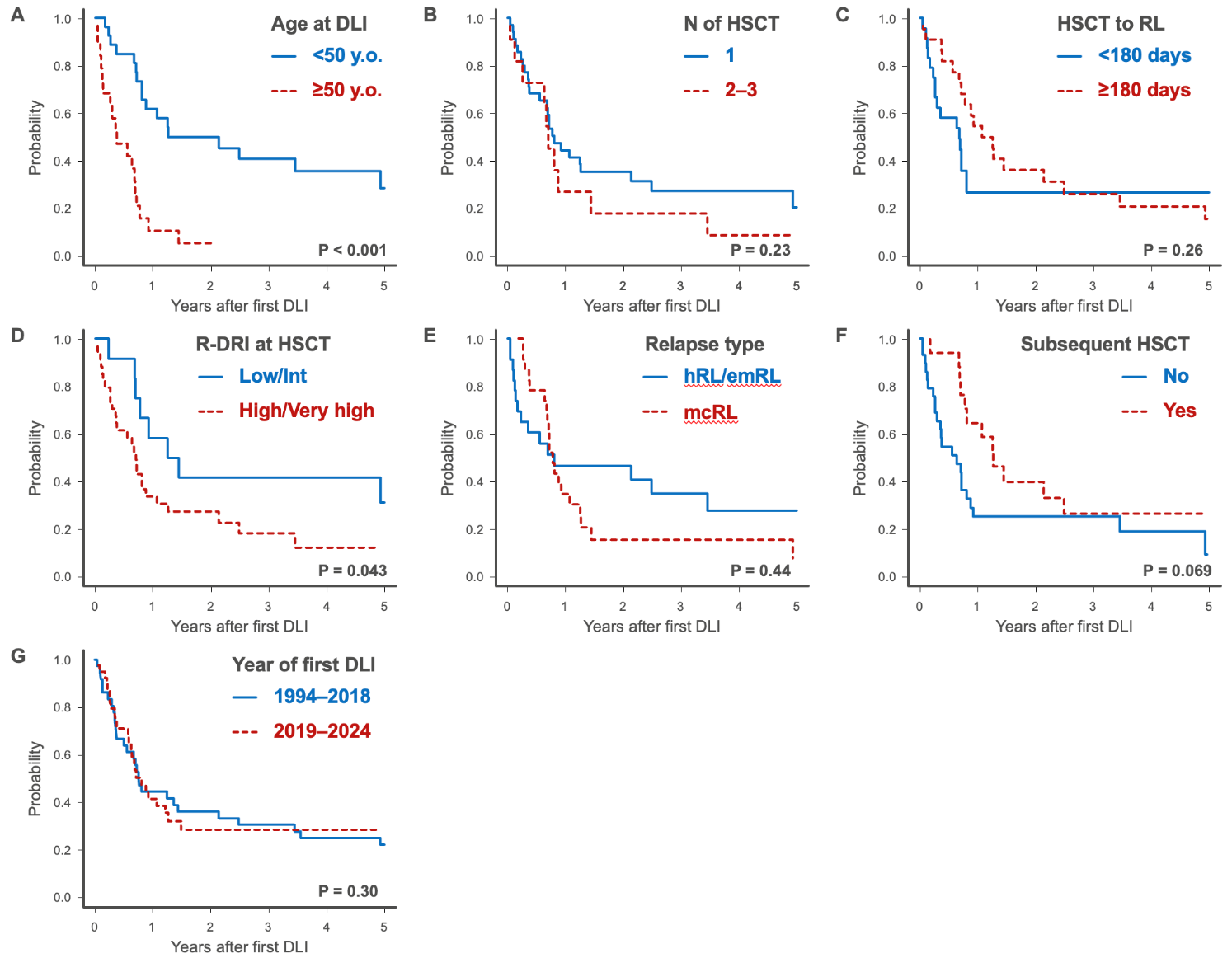


**Supplementary Figure 2. Kaplan-Meier survival curves for patients with acute myeloid leukemia receiving donor lymphocyte infusion**

Kaplan-Meier survival curves of the 46 patients with AML who underwent DLI. The plots show overall survival stratified by various factors and the log-rank test was used to assess any significant differences: age at first DLI (<50 versus ≥50 years, **A**), number of HSCT before DLI (1 versus 2–3, **B**), interval from HSCT to relapse (<180 days versus ≥180 days, **C**), R-DRI at HSCT (Low/Intermediate versus High/Very high, **D**), relapse type (hRL/emRL versus mcRL, **E**), receiving subsequent HSCT (Yes versus No, **F**), and year of first DLI (1994–2018 versus 2019–2024, **G**).

AML, acute myeloid leukemia; DLI, donor lymphocyte infusion; emRL, extramedullary relapse; HSCT, hematopoietic stem cell transplantation; hRL, hematological relapse; mcRL, molecular or cytogenetic relapse; N, number; R-DRI, refined disease risk index; RL, relapse.

**
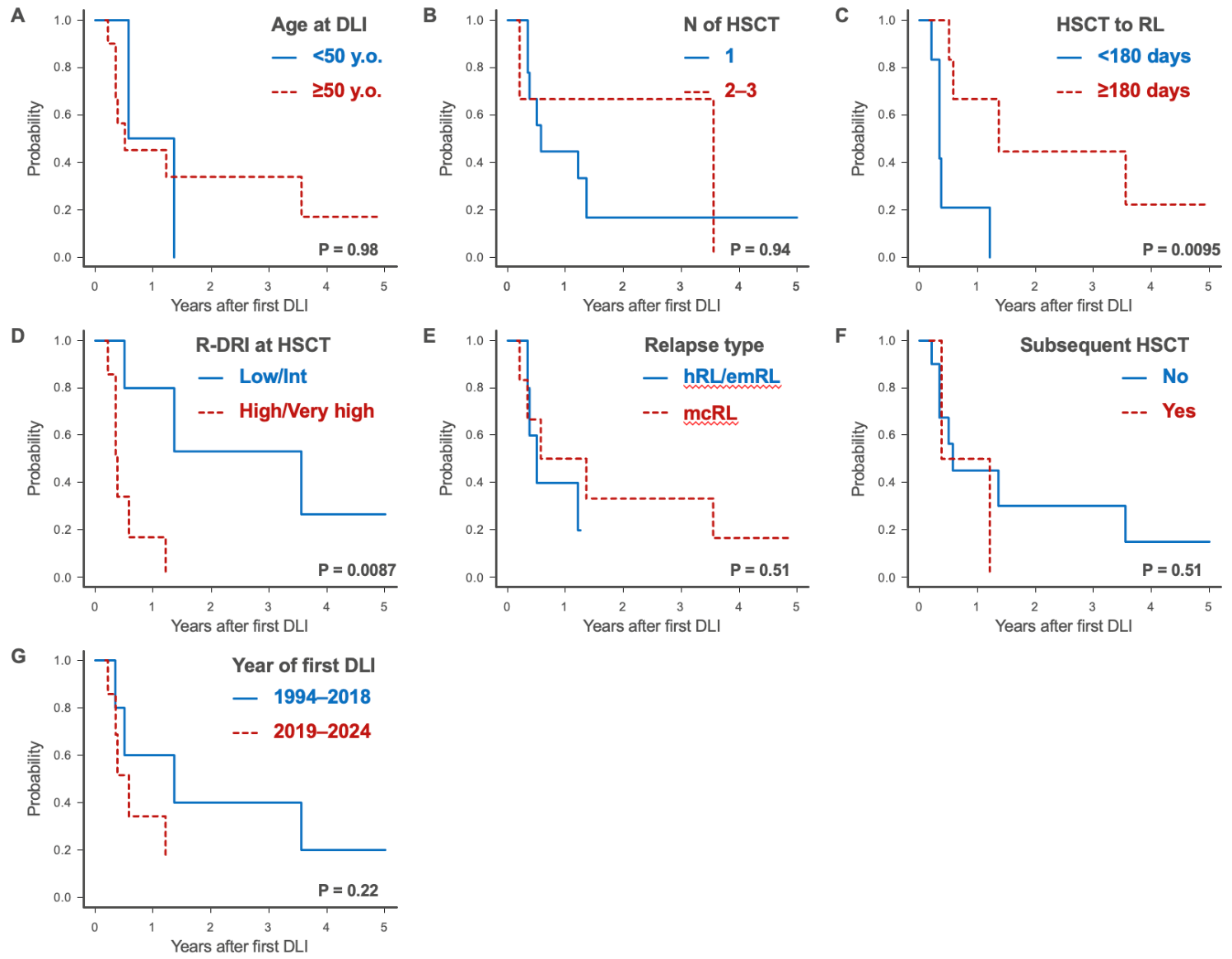
**

**Supplementary Figure 3. Kaplan–Meier survival curves for patients with myelodysplastic syndromes receiving donor lymphocyte infusion**

Kaplan-Meier survival curves of the 12 patients with MDS who underwent DLI. The plots show overall survival stratified by various factors and the log-rank test was used to assess any significant differences: age at first DLI (<50 versus ≥50 years, **A**), number of HSCT before DLI (1 versus 2–3, **B**), interval from HSCT to relapse (<180 days versus ≥180 days, **C**), R-DRI at HSCT (Low/Intermediate versus High/Very high, **D**), relapse type (hRL/emRL versus mcRL, **E**), receiving subsequent HSCT (Yes versus No, **F**), and year of first DLI (1994–2018 versus 2019–2024, **G**).

DLI, donor lymphocyte infusion; emRL, extramedullary relapse; HSCT, hematopoietic stem cell transplantation; hRL, hematological relapse; mcRL, molecular or cytogenetic relapse; MDS, myelodysplastic syndromes; N, number; R-DRI, refined disease risk index; RL, relapse.

**
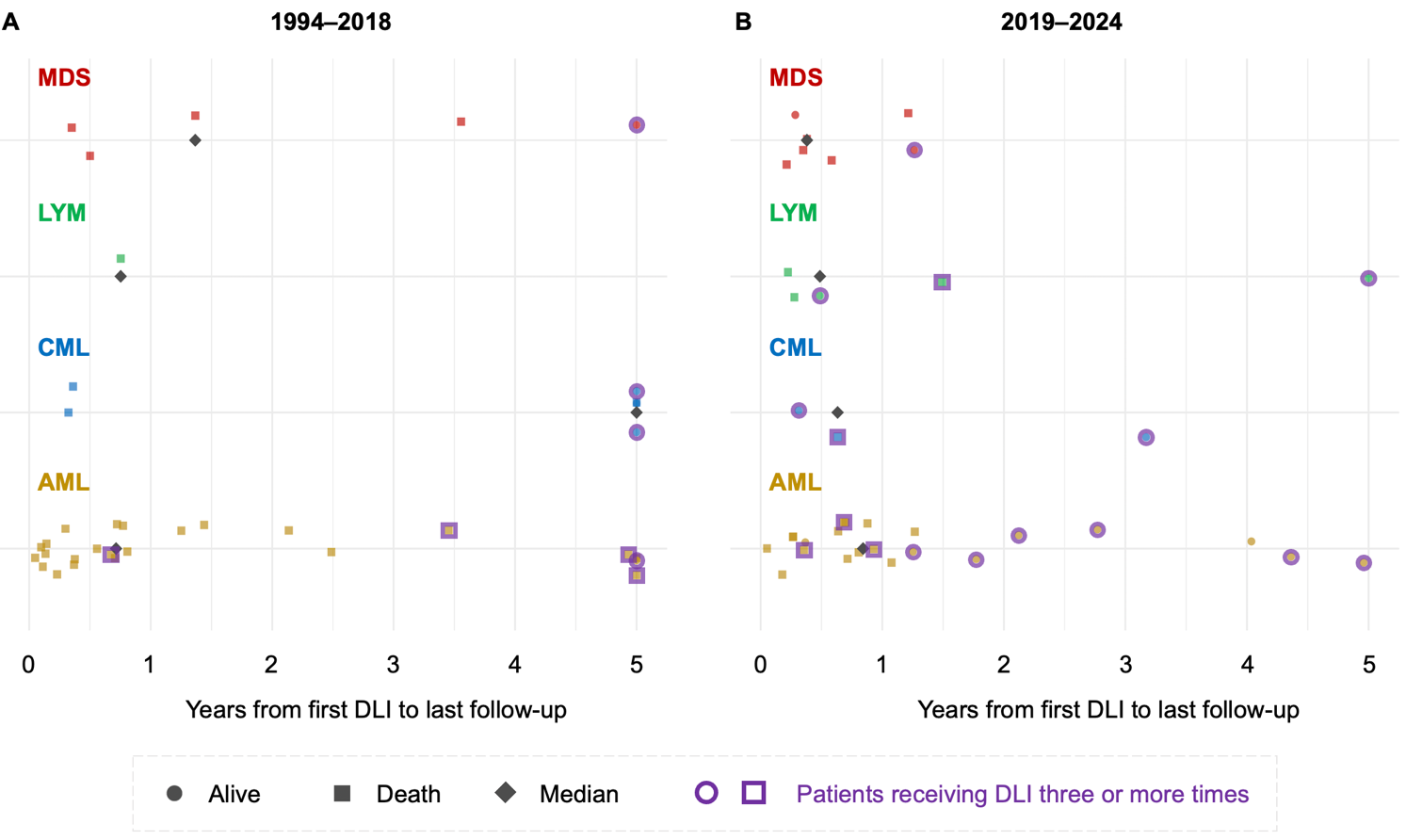
**

**Supplementary Figure 4. Dot plots of survival duration in patients who received donor lymphocyte infusion**

The first group consisted of 36 cases from 1994 to 2018 (**A**), and the second group included 39 cases from 2019 to 2024 (**B**). The x-axis represents years from the first DLI to the last follow-up. Patients alive at the last follow-up are shown as circles, while deceased patients are represented by squares. Patients who received three or more DLIs are highlighted in purple circles or squares. Median survival durations for each disease group are indicated by diamonds. Patients with a follow-up period exceeding five years are plotted at the 5-year mark.

AML, acute myeloid leukemia; CML, chronic myeloid leukemia; DLI, donor lymphocyte infusion; LYM, lymphoid malignancies; MDS, myelodysplastic syndromes.

## Supplementary Tables

**Supplementary Table 1. Baseline characteristics stratified by year of donor lymphocyte infusion**

| Patient characteristics |  | 1994–2018 | 2019–2024 | *P* |
| --- | --- | --- | --- | --- |
| N (%) |  | 36 (100.0) | 39 (100.0) |  |
| Age at first DLI | Years, median [range] | 45 [20–69] | 51 [24–69] | 0.12 |
| Sex | Female | 13 (36.1) | 16 (41.0) | 0.81 |
|  | Male | 23 (63.9) | 23 (59.0) |  |
| Underlying disease | AML | 24 (66.7) | 22 (56.4) | 0.25 |
|  | MDS | 5 (13.9) | 7 (17.9) |  |
|  | CML | 6 (16.7) | 3 (7.7) |  |
|  | MPN | 0 (0.0) | 2 (5.1) |  |
|  | ALL/LBL | 0 (0.0) | 4 (10.3) |  |
|  | ML | 1 (2.8) | 1 (2.6) |  |
| R-DRI at HSCT | Low | 5 (13.9) | 2 (5.1) | 0.16 |
|  | Intermediate | 11 (30.6) | 12 (30.8) |  |
|  | High | 14 (38.9) | 23 (59.0) |  |
|  | Very high | 6 (16.7) | 2 (5.1) |  |
| Year of HSCT | Years, median [range] | 2012 [1992–2018] | 2020 [2017–2023] | <0.001 |
| Numbers of HSCT before DLI | 1 | 28 (77.8) | 31 (79.5) | 0.88 |
|  | 2 | 7 (19.4) | 8 (20.5) |  |
|  | 3 | 1 (2.8) | 0 (0.0) |  |
| Graft source | HLA-matched related BM | 8 (22.2) | 3 (7.7) | 0.042 |
|  | HLA-matched related PBSC | 9 (25.0) | 4 (10.3) |  |
|  | Unrelated BM | 13 (36.1) | 18 (46.2) |  |
|  | Unrelated PBSC | 0 (0.0) | 4 (10.3) |  |
|  | HLA-haploidentical related PBSC | 6 (16.7) | 10 (25.6) |  |
| Conditioning intensity | Myeloablative | 26 (72.2) | 16 (41.0) | 0.010 |
|  | Reduced intensity | 10 (27.8) | 23 (59.0) |  |
| Interval from HSCT to relapse | Days, median [range] | 37 [7–190] | 51 [11–443] | 0.22 |
| Interval from relapse to first DLI | Days, median [range] | 223 [46–2,688] | 159 [21–825] | 0.18 |
| Relapse type | Hematological relapse | 18 (50.0) | 14 (35.9) | 0.32 |
|  | Extramedullary relapse | 2 (5.6) | 6 (15.4) |  |
|  | Molecular or cytogenetic relapse | 16 (44.4) | 19 (48.7) |  |
| Numbers of DLI | 1 | 16 (44.4) | 14 (35.9) | 0.21 |
|  | 2 | 12 (33.3) | 9 (23.1) |  |
|  | ≥3 | 8 (22.2) | 16 (41.0) |  |
| Infused CD3-positive cells | Initial dose, ×10^7^ cells/kg,  median [range] | 1.07  [0.10–8.45] | 0.14  [0.06–2.00] | <0.001 |
|  | Mean dose per DLI, ×10^7^ cells/kg,  median [range] | 1.31  [0.10–8.45] | 0.37  [0.06–3.56] | <0.001 |
|  | Total dose, ×10^7^ cells/kg,  median [range] | 2.16  [0.11–29] | 0.72  [0.06–17.78] | 0.019 |
| Combination therapy with DLI | Azacitidine only | 7 (19.4) | 11 (28.2) | 0.006 |
|  | Azacitidine and venetoclax | 0 (0.0) | 10 (25.6) |  |
|  | Azacitidine and FLT3 inhibitor | 0 (0.0) | 1 (2.6) |  |
|  | Azacitidine and gemtuzumab ozogamicin | 4 (12.9) | 0 (0.0) |  |
|  | Azacitidine, venetoclax, and FLT3 inhibitor | 0 (0.0) | 1 (2.6) |  |
|  | FLT3 inhibitor only | 0 (0.0) | 5 (12.8) |  |
|  | Venetoclax only | 0 (0.0) | 1 (2.6) |  |
|  | TKI only | 2 (5.6) | 1 (2.6) |  |
|  | TKI and other chemotherapy | 1 (2.8) | 1 (2.6) |  |
|  | Asciminib | 0 (0.0) | 1 (2.6) |  |
|  | Other cytotoxic chemotherapy only | 11 (30.6) | 5 (12.8) |  |
|  | Steroid, interferon, radiotherapy, or tretinoin | 4 (11.1) | 1 (2.6) |  |
|  | DLI only | 7 (19.4) | 1 (2.6) |  |
| Receiving subsequent HSCT | Yes | 13 (36.1) | 14 (35.9) | 1.00 |
|  | No | 23 (63.9) | 25 (64.1) |  |

ALL, acute lymphoblastic leukemia; AML, acute myeloid leukemia; BM, bone marrow; CML, chronic myeloid leukemia; DLI, donor lymphocyte infusion; HLA, human leukocyte antigen; HSCT, hematopoietic stem cell transplantation; LBL, lymphoblastic lymphoma; MDS, myelodysplastic syndromes; ML, malignant lymphoma; MPN, myeloproliferative neoplasms; N, number; PBSC, peripheral blood stem cells; R-DRI, refined disease risk index; TKI, tyrosine kinase inhibitor.

**Supplementary Table 2. Univariate analysis for overall survival in the entire cohort**

| Factor | Group | N | HR (95% CI) | *P* |
| --- | --- | --- | --- | --- |
| Age at first DLI | <50 years | 38 | 1 |  |
|  | ≥50 years | 37 | 2.15 (1.25–3.67) | 0.0053 |
| Sex | Female | 29 | 1 |  |
|  | Male | 46 | 0.73 (0.43–1.25) | 0.25 |
| R-DRI | Low or Intermediate | 30 | 1 |  |
|  | High or Very high | 45 | 3.07 (1.67–5.63) | <0.001 |
| Year of HSCT | 1992–2017 | 36 | 1 |  |
|  | 2018–2023 | 39 | 0.96 (0.56–1.66) | 0.89 |
| Numbers of HSCT before DLI | 1 | 59 | 1 |  |
|  | 2 or 3 | 16 | 1.87 (1.03–3.41) | 0.039 |
| Interval from HSCT to relapse | <180 days | 38 | 1 |  |
|  | ≥180 days | 37 | 0.49 (0.28–0.84) | 0.0097 |
| Relapse type | Hematological or extramedullary relapse | 40 | 1 |  |
|  | Molecular or cytogenetic relapse | 35 | 1.00 (0.59–1.7) | 0.99 |
| Year of first DLI | 1994–2018 | 36 | 1 |  |
|  | 2019–2024 | 39 | 1.00 (0.58–1.72) | 0.99 |

DLI, donor lymphocyte infusion; HR, hazard ratio; HSCT, hematopoietic stem cell transplantation; N, number; R-DRI, refined disease risk index.

**Supplementary Table 3. Baseline characteristics in patients with acute myeloid leukemia and myelodysplastic syndromes**

| Patient characteristics |  | AML | MDS | *P* |
| --- | --- | --- | --- | --- |
| N (%) |  | 46 (100.0) | 12 (100.0) |  |
| Age at first DLI | Years, median [range] | 47 [20–69] | 58 [26–69] | 0.018 |
| Sex | Female | 22 (47.8) | 1 (8.3) | 0.019 |
|  | Male | 24 (52.2) | 11 (91.7) |  |
| R-DRI at HSCT | Low | 3 (6.5) | 0 (0.0) | 0.32 |
|  | Intermediate | 9 (19.6) | 5 (41.7) |  |
|  | High | 29 (63.0) | 7 (58.3) |  |
|  | Very high | 5 (10.9) | 0 (0.0) |  |
| Year of HSCT | Years, median [range] | 2017 [1998–2023] | 2020 [2011–2023] | 0.22 |
| Numbers of HSCT before DLI | 1 | 35 (76.1) | 9 (75.0) | 1.00 |
|  | 2 | 10 (21.7) | 3 (25.0) |  |
|  | 3 | 1 (2.2) | 0 (0.0) |  |
| Graft source | HLA-matched related BM | 5 (10.9) | 1 (8.3) | 0.21 |
|  | HLA-matched related PBSC | 10 (21.7) | 1 (8.3) |  |
|  | Unrelated BM | 16 (34.8) | 9 (75.0) |  |
|  | Unrelated PBSC | 3 (6.5) | 0 (0.0) |  |
|  | HLA-haploidentical related PBSC | 12 (26.1) | 1 (8.3) |  |
| Conditioning intensity | Myeloablative | 28 (60.9) | 5 (41.7) | 0.33 |
|  | Reduced intensity | 18 (39.1) | 7 (58.3) |  |
| Interval from HSCT to relapse | Days, median [range] | 175 [21–1740] | 202 [28–721] | 0.63 |
| Interval from relapse to first DLI | Days, median [range] | 37 [7–443] | 67 [29–149] | 0.02 |
| Relapse type | Hematological relapse | 2 (4.3) | 0 (0.0) | 1.00 |
|  | Extramedullary relapse | 21 (45.7) | 6 (50.0) |  |
|  | Molecular or cytogenetic relapse | 23 (50.0) | 6 (50.0) |  |
| Numbers of DLI | 1 | 19 (41.3) | 4 (33.3) | 0.40 |
|  | 2 | 13 (28.3) | 6 (50.0) |  |
|  | ≥3 | 14 (30.4) | 2 (16.7) |  |
| Infused CD3-positive cells | Initial dose, ×10^7^ cells/kg,  median [range] | 0.50 [0.06-8.45] | 0.30 [0.08-2.11] | 0.63 |
|  | Mean dose per DLI, ×10^7^ cells/kg,  median [range] | 0.94 [0.06–8.45] | 0.57 [0.11–2.66] | 0.70 |
|  | Total dose, ×10^7^ cells/kg,  median [range] | 1.15 [0.06–17.78] | 0.99 [0.11–8.84] | 0.46 |
| Combination therapy with DLI | Azacitidine only | 10 (21.7) | 8 (66.7) | 0.12 |
|  | Azacitidine and venetoclax | 7 (15.2) | 3 (25.0) |  |
|  | Azacitidine and FLT3 inhibitor | 1 (2.2) | 0 (0.0) |  |
|  | Azacitidine and gemtuzumab ozogamicin | 4 (8.7) | 0 (0.0) |  |
|  | Azacitidine, venetoclax, and FLT3 inhibitor | 1 (2.2) | 0 (0.0) |  |
|  | FLT3 inhibitor only | 5 (10.9) | 0 (0.0) |  |
|  | Venetoclax only | 1 (2.2) | 0 (0.0) |  |
|  | Other cytotoxic chemotherapy only | 9 (19.6) | 1 (8.3) |  |
|  | Steroid | 1 (2.2) | 0 (0.0) |  |
|  | Radiotherapy | 2 (4.3) | 0 (0.0) |  |
|  | DLI only | 5 (10.9) | 0 (0.0) |  |
| Receiving subsequent HSCT after DLI | Yes | 17 (37.0) | 2 (16.7) | 0.30 |
|  | No | 29 (63.0) | 10 (83.3) |  |
| Year of first DLI |  | 2018 [1999–2024] | 2020 [2012–2023] | 0.30 |

AML, acute myeloid leukemia; BM, bone marrow; DLI, donor lymphocyte infusion; HLA, human leukocyte antigen; HSCT, hematopoietic stem cell transplantation; MDS, myelodysplastic syndromes; N, number; PBSC, peripheral blood stem cells; R-DRI, refined disease risk index.

**Supplementary Table 4. Univariate analysis for overall survival in patients with acute myeloid leukemia**

| Factor | Group | N | HR (95% CI) | *P* |
| --- | --- | --- | --- | --- |
| Age at first DLI | <50 years | 27 | 1 |  |
|  | ≥50 years | 19 | 4.37 (2.09–9.12) | <0.001 |
| Sex | Female | 22 | 1 |  |
|  | Male | 24 | 0.70 (0.36–1.35) | 0.28 |
| R-DRI | Low or Intermediate | 12 | 1 |  |
|  | High or Very high | 34 | 2.26 (1.01–5.07) | 0.049 |
| Year of HSCT | 1992–2017 | 24 | 1 |  |
|  | 2018–2023 | 22 | 0.65 (0.33–1.29) | 0.22 |
| Numbers of HSCT before DLI | 1 | 35 | 1 |  |
|  | 2 or 3 | 11 | 1.55 (0.76–3.15) | 0.23 |
| Interval from HSCT to relapse | <180 days | 24 | 1 |  |
|  | ≥180 days | 22 | 0.68 (0.35–1.32) | 0.26 |
| Relapse type | Hematological or extramedullary relapse | 23 | 1 |  |
|  | Molecular or cytogenetic relapse | 23 | 1.31 (0.66–2.59) | 0.44 |
| Year of first DLI | 1994–2018 | 24 | 1 |  |
|  | 2019–2024 | 22 | 0.70 (0.35–1.38) | 0.30 |

DLI, donor lymphocyte infusion; HR, hazard ratio; HSCT, hematopoietic stem cell transplantation; N, number; R-DRI, refined disease risk index.

**Supplementary Table 5. Univariate analysis for overall survival in patients with myelodysplastic syndromes**

| Factor | Group | N | HR (95% CI) | *P* |
| --- | --- | --- | --- | --- |
| Age at first DLI | <50 years | 2 | 1 |  |
|  | ≥50 years | 10 | 0.98 (0.19–4.93) | 0.98 |
| Sex | Female | 1 | 1 |  |
|  | Male | 11 | 0.90 (0.11–7.62) | 0.93 |
| R-DRI | Low or Intermediate | 5 | 1 |  |
|  | High or Very high | 7 | 10.54 (1.22–91.24) | 0.033 |
| Year of HSCT | 1992–2017 | 5 | 1 |  |
|  | 2018–2023 | 7 | 2.66 (0.51–13.81) | 0.25 |
| Numbers of HSCT before DLI | 1 | 9 | 1 |  |
|  | 2 or 3 | 3 | 1.06 (0.20–5.53) | 0.94 |
| Interval from HSCT to relapse | <180 days | 8 | 1 |  |
|  | ≥180 days | 4 | 0.14 (0.03–0.77) | 0.024 |
| Relapse type | Hematological or extramedullary relapse | 6 | 1 |  |
|  | Molecular or cytogenetic relapse | 6 | 0.61 (0.14–2.77) | 0.53 |
| Year of first DLI | 1994–2018 | 5 | 1 |  |
|  | 2019–2024 | 7 | 2.66 (0.51–13.81) | 0.25 |

DLI, donor lymphocyte infusion; HR, hazard ratio; HSCT, hematopoietic stem cell transplantation; N, number; R-DRI, refined disease risk index.
